# Fistulous Withers Treated by Actual Cautery

**Published:** 1896-10

**Authors:** James A. Waugh

**Affiliations:** Pittsburg, Pa.


### FISTULOUS WITHERS TREATED BY ACTUAL CAUTERY.

By JAMES A. WAUGH, V.S.,
PITTSBURG, PA.

A Sewickley liveryman called me to treat a sorrel saddle-horse whose withers had been injured by a lady's side-saddle; then the injured parts were blistered twice and the patient was turned out to pasture for three months, but was returned uncured. A medical doctor was present when I appeared at the stable, and he generously volunteered information how they would treat a similar case in human practice. Then I kindly stated that this subject should and would be treated on the same general principles.

The owner insisted that he did not want his horse butchered and scarred up like what he had lately seen in a local veterinarian's infirmary. Therefore, I probed the fistulous sinuses along both sides of the withers, and found a very large abscess-cavity under the first layer of muscles in the neck under the part where the harness-collar pad bears at work; then made small incision in most dependent parts of abscess-cavity, syringed out cleanly; then packed this with marine lint well saturated in carbolic acid, sewed up all openings, and had dressings removed in thirty-six hours, and thereafter had wound dressed thrice daily with creolin lotion applied with a hard-rubber bulb-syringe with continuous flow. (California veterinarians taught me the benefits derived from the syringe.) However, the case did not progress as rapidly as desired, and I finally decided to test the actual cautery in this particular case. I had often secured favorable results from the use of the actual cautery in the treatment of chronic and obstinate quittors, and considered this a good case to test and report results.

We took the patient to a blacksmith shop, heated and pointed a horseshoe-bar into a suitable firing-iron, applied a twitch to upper lip as a means of restraint, then plunged the hot iron into the old incisions and clear through the abscess-cavity and out through the other side, and held it in position about forty-five seconds, then repeated once in a few minutes. This made two large holes on each side and through the abscess-cavity, which admitted free and open drainage. I did not see the patient for some time, but owner informed me that the old abscess or tumor swelled fearfully for a few days, and was about the size of a farmer's tea-kettle. Creolin lotion was used twice daily, and wounds were well washed out by means of rubber bulb-syringe with continuous flow. Result, rapid and satisfactory recovery with only very small scars scarcely visible.

It must be remembered that fine horses are highly prized and their owners are much opposed to any operations that are liable to leave unsightly scars or blemishes or even marks that might attract attention on the public highways.

I have had considerable experience in treating fistulous withers in cavalry horses, and had opportunity to observe that various lotions prove successful when carefully applied, while they generally fail if neglected or even applied carelessly in practice. I have often thought that we could derive great benefit in treating fistulous withers by confining our patients in a Trevis sling, same as described in Prof. James Law's *Farmer's Veterinary Adviser*, and at same time confine the head short and secure, and supply food and water at a fixed elevation to limit action of head, neck, shoulders, and fore-limbs. I intend to test this plan, and perhaps devise a useful and modern method that will hasten recovery.

